# Enablers and barriers to the implementation of the Food System Transformative Integrated Policy (FS-TIP) in Ghana, Malawi and Rwanda

**DOI:** 10.1186/s12889-026-28169-4

**Published:** 2026-08-01

**Authors:** Antonina N. Mutoro, Elizabeth Wambui, Ajibola Ogunsola, Dickson Amugsi, Gershim Asiki, Elizabeth W. Kimani-Murage

**Affiliations:** African Population and Health Research Center (APHRC), Nairobi, Kenya

**Keywords:** Food systems, Policy, Africa, Policy transformation, Food insecurity

## Abstract

**Background:**

The Food System Transformative Integrated Policy (FS-TIP) is an evidence-based framework developed to identify key food system challenges, relevant policies, and the actors necessary to support food systems. Following pilot implementation in Ghana, Malawi, and Rwanda, this study evaluated the initiative by exploring perceptions of its relevance, stakeholder preparedness to implement the approach, the degree of stakeholder involvement, and the enablers and barriers influencing implementation.

**Methods:**

This was a cross-sectional mixed methods study conducted in Ghana, Malawi and Rwanda. Data on willingness to adopt the process and perceived relevance of the initiative was collected using an online semi-structured questionnaire and key informant interviews were conducted to capture enablers and barriers. To assess the level of multi-stakeholder engagement, a desk review of process outputs was also conducted.

**Results:**

Ten stakeholders (Ghana = 2, Malawi = 5, Rwanda = 3) completed the online survey, and twenty-five participants (Ghana = 10, Malawi = 6, Rwanda = 7, project management team = 2) took part in interviews. Respondents reported willingness to adopt FS-TIP, perceived it as relevant to the African context, and considered it policy-oriented, though challenging to implement. Facilitators of implementation included stakeholder buy-in, involvement of local experts, and access to routinely collected data, whereas barriers included limited centralized databases, restricted stakeholder engagement, and inadequate implementation time.

**Conclusion:**

The FS-TIP initiative identified key food system challenges in Ghana, Malawi, and Rwanda. Stakeholders viewed the approach as relevant, acceptable, and feasible for adoption, with the potential to strengthen food system diagnostics and policy development. To promote long-term ownership and sustainability, the initiative would ideally be government-led and embedded within existing structures and initiatives.

**Supplementary Information:**

The online version contains supplementary material available at https://doi.org/10.1186/s12889-026-28169-4.

## Introduction

Food systems across the globe are currently facing multifaceted challenges, with the African continent disproportionately affected. Africa is simultaneously battling the persistent scourge of food insecurity which affects over 20% of the population [23] and the pervasive impact of the nutrition transition, a phenomenon characterized by rapidly shifting dietary patterns across its diverse populations [19]. Unhealthy diets contribute to nearly one in five deaths worldwide, and Africa is home close to half of the 20 countries experiencing a rise in adult obesity rates [3]. Undernutrition also remains prevalent with 282 million people undernourished [6]. This imbalance is characterized by the high of harmful foods and the low consumption of nutritious foods. Apart from access to food and food consumption patterns, issues on soil health, local production patterns and management of food loss and waste remain pertinent [12]. This highlights the need for transformation of African food systems which can only be achieved with better coordinated action across different sectors to ensure that policies and interventions are aligned and effective [11, 14].

Transforming how the food system runs, especially from a policy perspective, can lead to food system transformation. Current governance mechanisms are siloed limiting: participation of different actors mostly vulnerable groups, transparency, and accountability. This hinders comprehensive change, execution and monitoring of food systems [18] OECD 2021). Applying interdisciplinary and transdisciplinary approaches to understand power dynamics in food systems can help identify and explain existing inequalities [2, 11]. Participatory approaches, such as intersectoral working groups, multi-stakeholder policy dialogues, and consultative processes, can further support identification of context-specific priorities, enhance data sharing, and increase alignment between national strategies and local implementation (McQueen et al. 2012; [11].

Policy changes to transform and strengthen food systems have been limited, especially in low and middle-income countries, partly because of gaps in data required to monitor and evaluate different food systems components, the lack of proper multi sectoral coordination mechanisms and investment into food systems transformation [9, 12, 14]. These gaps limit the ability to develop, implement and scale up effective and sustainable policies (OECD, 2021; [2].

Countries such as Ghana, Malawi and Rwanda have made significant strides in food system transformation by developing national food systems strategies that are aligned regional initiatives such as the Malabo Declaration and the Comprehensive Africa Agriculture Development Programme (CAADP) [3, 9]. Despite this, there are existing gaps that limit food system transformation efforts including limited investment in Agriculture, lack of data on different aspects such as agricultural production, food loss and waste, impact of climate change of the food system, lack of infrastructure.

The Food System Transformative Integrated Policy (FS-TIP) initiative was developed to to address some of these gaps. The initiative was led by the project management office which included the following organizations: the African Population Health Research Centre, AKADEMIYA2063, Alliance for a Green Revolution in Africa, Boston Consulting Group, International Development Research Centre, International Food Policy Research Institute, Rockefeller Foundation, Tony Blair Institute for Global Change and the World Food Programme [9]. FS-TIP was rolled out to support the commitment of these three governments to developing and implementing transformative and integrated food systems policies. The analysis identified relevant food system stakeholders, existing food-related policies, and evidence gaps in Ghana, Malawi and Rwanda and was contextualized to each country’s needs and priorities. It was implemented in three phases: Phase 1 involved a landscape and diagnostic analysis of the current state of the food system; Phase 2 involved the design and implementation of food system policies and phase 3 would involve scale up of the same processes in other African countries.

The first phase, conducted from April and September 2021, focused on mapping stakeholders to identify key actors within the food system and clarify their respective roles. It also included a policy review to examine food system–related policies and identify implementation gaps, as well as indicator mapping to determine potential measures for tracking food system changes and identifying evidence deficiencies [10]. The process was coordinated by the project management office, with support from country-based experts engaged as consultants to undertake landscape assessments and diagnostic analyses. Stakeholders within participating countries contributed relevant information and documentation and were involved in validating the resulting outputs. The initiative built and leveraged on the momentum and global timing of the UN Food Systems Summit for mobilization and visibility, but has its sights firmly set on a multi-year journey that extends well beyond the Summit towards the fulfilment of the 2030 Sustainable Development Goals agenda. It brings together a strong coalition of organizations deeply committed to Africa’s sustainable and equitable development towards nourishing, regenerative, resilient and inclusive food systems. The initiative is guided by 11 principles as shown in supplementary Table 1.

Because the FS-TIP initiative represents an innovative approach to integrated food systems policy development, there was a need to systematically evaluate both the process through which it was implemented and its potential for adoption in different country contexts. This study is guided by implementation and governance theories to better understand how multi-sectoral food systems initiatives are designed, operationalized, and taken up by stakeholders. Specifically, the study draws on the Consolidated Framework for Implementation Research (CFIR) and the Intersectoral Governance Framework to examine stakeholder engagement, perceived relevance and usefulness of the FS-TIP approach, and the institutional and coordination mechanisms shaping its adoption (Damschroder et al. 2009; McQueen et al. 2012). Guided by these frameworks, the study aimed to evaluate: (1) the extent of multi-sectoral stakeholder participation in FS-TIP activities; and (2) stakeholder perceptions of the relevance, usefulness, acceptability, willingness, and preparedness to adopt the FS-TIP approach, alongside barriers and enabling factors influencing its successful implementation.

### Theoretical frameworks

This study was informed by two complementary theoretical frameworks: the Consolidated Framework for Implementation Research (CFIR) and the Intersectoral Governance Framework. The Consolidated Framework for Implementation Research (CFIR) offers a comprehensive structure for identifying factors that influence the implementation and adoption of complex interventions across diverse contexts (Damschroder et al. 2009). In the context of FS-TIP, CFIR was particularly relevant for examining how stakeholders perceive the relevance, usefulness, and feasibility of the initiative, as well as the enablers and barriers influencing its uptake within different country settings. By applying CFIR, the study systematically captured both individual- and system-level factors shaping the implementation of integrated food systems policy approaches in Ghana, Malawi, and Rwanda.

To complement CFIR’s focus on implementation, the Intersectoral Governance Framework was used to examine the multi-sectoral dimensions of the FS-TIP initiative (McQueen et al. 2012). Given the inherently cross-sectoral nature of food systems, this framework was well suited to analysing how different actors interact.

Applied together, the two frameworks allowed for a more comprehensive understanding of FS-TIP as both an implementation process and a governance intervention. Collectively, these frameworks guided the formulation of the study objectives, informed the selection of indicators and data collection tools, and shaped the interpretation of findings related to stakeholder engagement, adoption readiness, and the feasibility of scaling the FS-TIP approach to other contexts.

## Methods

### Study settings

The study was conducted in Ghana, Malawi and Rwanda. These countries were selected because they are on track towards achieving the Malabo Declaration on Accelerated Agricultural Growth by 2025 (African Union Commission and NEPAD 2014). The countries were also selected based on the degree of top-level government support, the existing alignment to the importance of creating transformative and integrated food policies, and the on-the-ground capabilities of the coalition (Table 1).

**Table 1:** A brief description of food systems in the selected countries. Sources: Laar et al. 2020; [3, 17]

Ghana	Malawi	Rwanda
In the 2018 Biennial Review, Ghana demonstrated strong performance in multi-sectoral and multi-stakeholder coordination, as well as in fostering inclusive public–private partnerships (PPPs) across commodity value chains. The country also showed notable progress in strengthening the resilience of farming, pastoralist, and fishing households. Additionally, Ghana ranked highly in efforts to generate employment opportunities for women and youth in agribusiness and throughout the food system..	Malawi ranked among the top ten performing countries in Africa across several CAADP commitments and targets and emerged as the leading performer in Southern Africa. The country significantly surpassed its target for youth employment in agricultural value chains, exceeding it by more than 200 percent Malawi only narrowly missed the targets on public expenditure on the agricultural sector, and was nearly on track to meet its commitments on budgetary allocation for social protection for vulnerable social groups	It performs exceedingly well on the quality of multi-sectoral and multi-stakeholder coordination and on evidence-based policies that are well supported at institutional level. Most importantly, Rwanda has also made much headway on improving productivity, reducing post-harvest loss, creating inclusive PPPs for commodity value chains and creating jobs for women and young people in the agricultural sector and across food systems. Specifically, the land consolidation and Integrated Development Program (IDP) policies were implemented to increase agricultural production and efficient land use along with stimulating job creation. While this has not translated into improved diets, Rwanda has seen increased household sales of agricultural products, particularly animal-based proteins

### Study design and population

The study was a multiple case study mixed methods implementation research evaluation. It involved the use of qualitative and quantitative methods. The study design and data collection tools were informed by the theoretical frameworks described above.

Stakeholders in the public, social and private sectors involved in food systems were included in the study. These were top-level government officials, non-governmental organizations, civil societies and industry actors. Participants for the study were purposively selected, paying particular attention to their knowledge, experience and participation in the process.

### Sampling strategy and sample size

The study was not a population level study and was not intended to be generalizable. Consequently, no formal sample size calculation was undertaken, as the sample was intended primarily for descriptive analysis. Purposive sampling was applied in both the quantitative and qualitative components, with participants selected based on their prior involvement in the FS-TIP process. For the quantitative component, stakeholder lists were obtained from country partners. Selection of qualitative participants was guided by factors such as institutional affiliation, level of participation in the process, relevant expertise and experience, and their perceived importance within food systems. All participants were contacted by email.

### Data collection

The key indicators of the study included: the perceived relevance of FS-TIP outputs to national FS challenges; stakeholder readiness to adopt FS-TIP outputs, including willingness and perceived capacity; level of food system stakeholder engagement in FS-TIP processes, measured by participation in meetings, consultations, and decision-making and enablers and barriers to implementation, including structural, relational, and informational factors. The indicators were not validated but were grounded in evidence from food system governance frameworks such as the Intersectoral Governance Framework (McQueen 2012). This approach ensured that the evaluation captured both perceived and observed dimensions of stakeholder engagement and adoption feasibility.

Qualitative data on country experiences during the project, particularly relating to potential adoption of the evidence-based policy formulation and integration, as well as potential barriers and facilitators to policy development and implementation in each country, were collected using interview guides (supplementary file 1). Key informant interviews were conducted virtually and in English by experienced members of the research team. The interviews were audio-recorded and transcribed verbatim to ensure completeness. Each interview was conducted by a moderator, assisted by a note-taker.

Quantitative data was also collected virtually using a self-administered, semi-structured questionnaire, which captured data on the relevance and appropriateness of the FS-TIP process and outputs (supplementary file 1). The questionnaire also assessed the relevance and usefulness of the evidence-base and tool kit generated by the implementers. With support from our in-country partners, the online questionnaires were sent to stakeholders to complete and return them to the research team. The respondents were provided adequate instructions and guidance to ensure clarity and accuracy of the information collected.

### Desk review

To complement the qualitative and quantitative findings, a desk review of project materials and outputs was undertaken, including stakeholder meeting minutes, reports, and the toolkit.

An inter-sectoral governance framework (McQueen, 2012) was applied to evaluate multi-sectoral engagement within the process. This involved examining governance arrangements at different levels, including central government, parliament, civil service institutions, and funded agencies, as well as mechanisms for collaboration with actors outside government. The aim was to better understand the extent to which governance structures supported policy development and implementation. In addition, we reviewed the institutional affiliations of experts from each country and examined the relevance and degree of participation among stakeholders involved in the FS-TIP process. These findings were subsequently compared with stakeholders identified as critical to the functioning of national food systems.

### Analysis

Quantitative data was analyzed using the statistical software, R, version 4.0.5. The characteristics of study participants and stakeholder perceptions were summarized using frequencies and proportions.

Country-specific qualitative audio files were transcribed verbatim, anonymized and stored in digital format (Microsoft Word compatible). Data was imported into the qualitative data management software NVIVO 12, for coding and analysis. The transcribed word files were imported into NVIVO 12 software (QSR International Pty Ltd, Don Caster, Victoria, Australia) for coding and further analysis. Data was coded by an external coder and a quality assessment of the coded data was conducted by the team. Final checks for consistency of the application of the codes was undertaken by a member of the research team.

Qualitative data from interviews and document reviews were analyzed thematically. An initial deductive codebook was developed based on the CFIR domains intervention characteristics, outer setting, inner setting, characteristics of individuals, and implementation process to organize data related to implementation experiences, barriers, and enablers. Inductive codes were then generated to capture emerging context-specific themes.

## Results

Of the 55 stakeholders who were approached to complete the online quantitative survey, only 10 (Malawi, *n* = 5; Ghana, *n* = 2; Rwanda, *n* = 3) responded (Supplementary Fig. 1). Most of the stakeholders who responded (*n* = 7) worked in government, nearly all the respondents worked in the agricultural sector (*n* = 8) and all of them had tertiary education. Six of them had more than 12 years in service.

A total of 25 key informant interviews were conducted across the three participating countries: ten in Ghana, seven in Rwanda and six in Malawi. The remaining two interviews were conducted with members of the FS-TIP project management office. The study participants were drawn from universities, government ministries or agencies and non-governmental or civil society organizations (NGOs/CSOs) in the respective countries (Table 2).

**Table 2:** Key informant interview participants’ demographic characteristics (*N*=25)

**Socio-economic and demographic characteristics**	**FS-TIP Countries**		
	**Rwanda**	**Malawi**	**Ghana**
Gender			
Male	4	3	6
Female	3	3	4
Affiliation			
Government	1	1	4
NGO/CSO	4	5	3
Research/Academia	2	-	3

Desk review of stakeholder mapping outputs provided an additional perspective on participation. While mapping exercises in each country identified a broad range of potential food-system stakeholders, only 30–50 percent of these were ultimately engaged in FS-TIP implementation (Table 3).

**Table 3:** Comparison between stakeholders involved in the FS-TIP process and those identified during the stakeholder mapping process

	**Rwanda **		**Malawi**		**Ghana**	
	Food system stakeholder types actually involved	Food system stakeholder types recommended	Food system stakeholder types actually involved	Food system stakeholder types recommended	Food system stakeholder types actually involved	Food system stakeholder types recommended
Government	7	23	11	21	10	23
NGO - International community and development organizations	10	28	5	15	4	19
Research	2	8	1	1	1	12
Academia	1	2	1	3	5	5
Private/Business entity	1	20	1	21	0	17
Civil Society	1	6	1	5	0	24
Media	0	5	0	5	0	6
Total number of stakeholder types in each country	**22**	**92**	**20**	**71**	**20**	**106**

### Level of multi-sectoral stakeholder involvement in FS-TIP activities

Stakeholders participated in the FS-TIP process through two primary mechanisms: (1) as in-country experts responsible for activities such as policy and evidence review and stakeholder mapping; and (2) in advisory roles involving the review and validation of outputs, including reports produced through the process. The project management office recruited in-country experts to support and lead major workstreams, including data diagnostics, policy analysis, and stakeholder mapping. Some components were also undertaken by international consultants affiliated with institutions within the project management office. Evidence from the desk review and key informant interviews showed that in-country experts mainly represented universities, research institutions, NGOs, civil society organizations, business entities, and government agencies, with Malawi being an exception where only NGO consultants were engaged (Supplementary Table 2).The stakeholders from each country who acted in an advisory role were largely government officers from different sectors such as agriculture, national development and planning, while a few were drawn from international NGOs (Supplementary Table 3). This inclusion of participants primarily from the government contrasted with the type of participants working as in-country experts during the process itself which saw more representation of stakeholders from different sectors (Table 3).

“We were consulted towards the end, not really in shaping the process from the start.” (KII, government official, Malawi).

The stakeholder mapping exercise identified a wider range of key food system actors, including sectors, ministries, government agencies, and international and community development organizations, than those involved in FS-TIP implementation. Notably, private sector entities, media organizations, and civil society groups were recognized as important stakeholders but were not engaged in the process. Likewise, while many universities and local and international research institutions were identified as relevant food system actors across countries, only a limited number participated in FS-TIP activities.

### Stakeholder perceptions on the relevance of the FS-TIP process to their countries

**Fig. 1:**
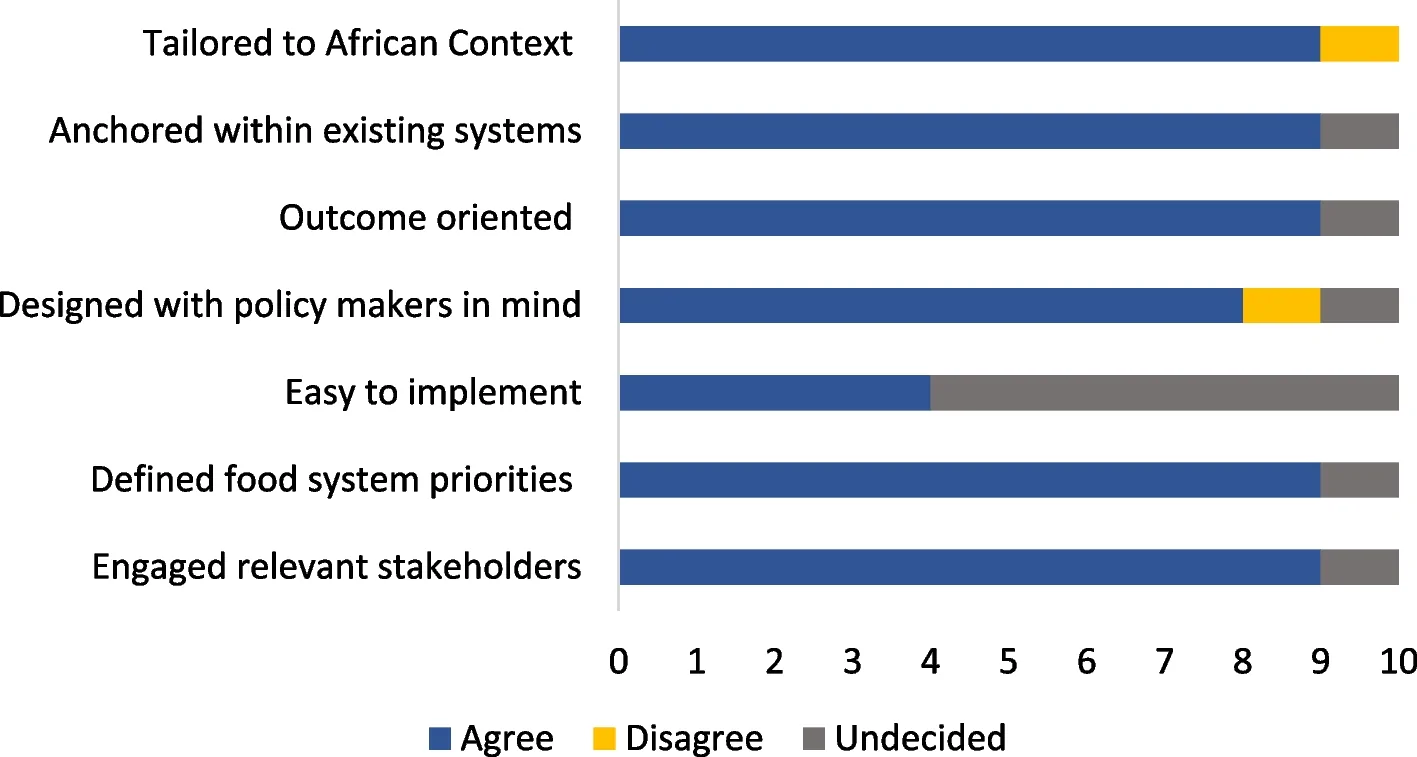
Perceptions on characteristics of the FS-TIP process based on stakeholder feedback (*N* = 10)

**Fig. 2:**
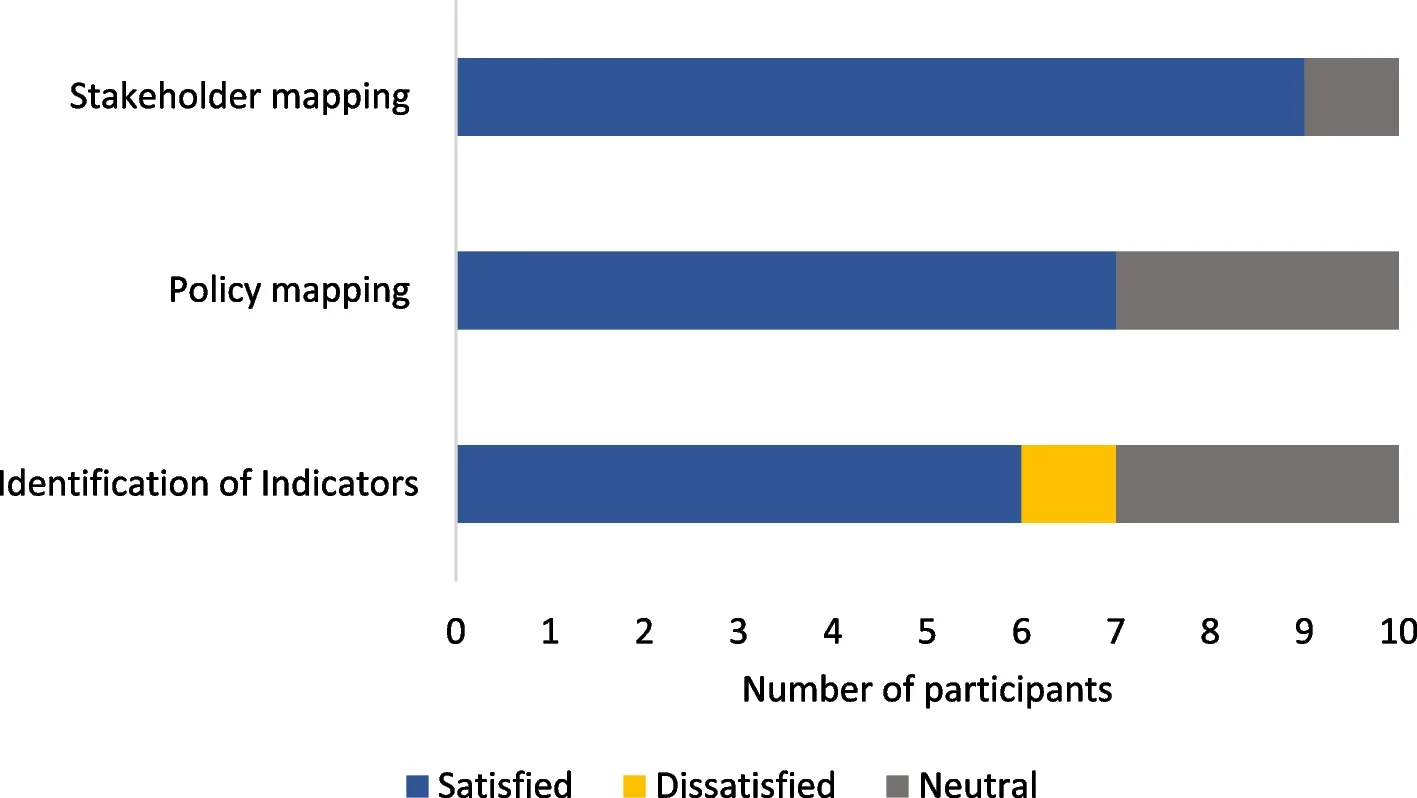
Stakeholder satisfaction with the FS-TIP process (*N* = 10)

Findings from the quantitative survey (Figs. 1 and 2) and KIIs indicated that all stakeholders perceived the FS-TIP process as relevant and aligned with national priorities. All stakeholders felt that the FS-TIP process was aligned with their country’s needs and was relevant and useful for their country. Respondents emphasized that FS-TIP contributed to identifying critical policy and data gaps and strengthened evidence for decision-making. Stakeholders from all three countries reported satisfaction with the main process components indicator selection, stakeholder mapping, and policy mapping and noted that the approach facilitated inter-sectoral understanding of food systems. Respondents felt that the stakeholder and policy mapping processes worked well given the short turnaround time.

Respondents particularly valued the indicator identification process, which they described as inclusive and informative. They noted that aligning indicators across sectors improved data sharing and allowed for more comprehensive analysis of food system challenges. Stakeholders in Malawi highlighted the alignment of FS-TIP outputs with national frameworks such as the Malawi 2063 Vision, while others cited the usefulness of the process in prioritizing context-specific food system issues.

**Fig. 3:**
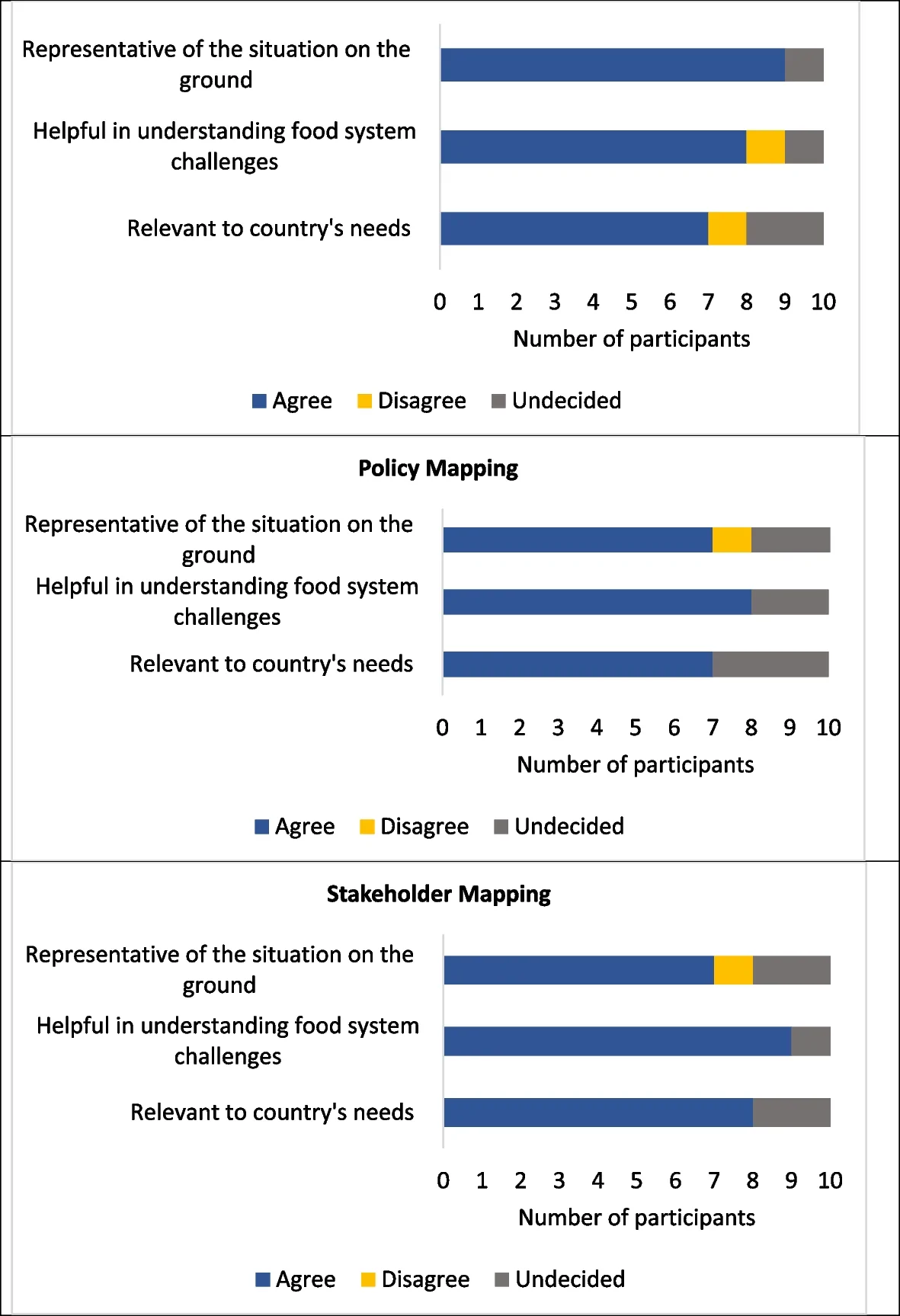
Perceptions on aspects of the FS-TIP processes (*N* = 10)

Stakeholders expressed willingness to adopt outputs generated through the FS-TIP process, particularly the identification of key indicators, as the process produced valuable evidence to inform policymaking, strengthen monitoring of nutrition-sensitive consumption and trade, and establish a shared framework for tracking progress (Fig. 3). Participants also supported adoption of outputs from the stakeholder mapping component, noting that engagement with key food system actors helped foster networks and had the potential to enhance coordination across the food system. In relation to policy mapping, stakeholders viewed the outputs favorably because they could help address implementation challenges within food systems and promote alignment and coherence among food-related policies (Fig. 3).

### Barriers to implementation of FS-TIP processes

Challenges in accessing the necessary data and information because of the lack of centralized databases containing food system resources in participating countries were reported. While all three countries had national statistical offices, they did not have all the required data. Experts often turned to other sources like scientific articles and reports to meet their data needs.

Conflicting reports and outdated sources further complicated efforts to establish a reliable evidence base. For example, some indicators, such as nutrient consumption, food loss, and waste, were either unavailable or reported inconsistently across sources, leaving experts uncertain about which data to rely on.

The scarcity of disaggregated data across various demographics (gender, age, region, or household) hindered the development of targeted interventions and the assessment of equity outcomes. This highlights the critical need for data systems that facilitate policy-relevant stratifications, especially in multi-sectoral food system initiatives. In cases where there were several related policy and strategy documents, experts found it difficult to determine which documents were informing policy implementation.

These barriers indicate the need for robust institutional and governance structures for the implementation of multi-sectoral policy tools like FS-TIP. Without functional data systems and early institutional buy-in, stakeholder willingness does not automatically lead to policy adoption or sustained implementation.

“We had to find data mainly online and the only challenge was sometimes we just couldn't find data on some indicators simply because those data were not collected in the country. And the indicators, they were selected for different countries so some indicators might have been available in other countries but not in Rwanda. So, I think that was the only challenge: not finding data for some indicators.” (KII, academic, Rwanda).

*“You find that there is some mismatch between data and you really don't know which one to take. But even apart from that, there are times when you could find two reports from public institutions that have different figures of the same data, and you could be asking what happened. In that case, you don't really know who to trust.”* (KII, academic, Rwanda).

### Enablers to adoption of systems approach in FS-TIP processes

#### Existing national data and information infrastructure

Availability of routine data from national surveys, such as the Demographic and Health Survey (DHS), agricultural censuses, and Rwanda’s Integrated Household Living Conditions Survey, provided a foundation for indicator assessment. These pre-existing datasets allowed FS-TIP to integrate into ongoing monitoring systems rather than creating parallel structures, enhancing feasibility and sustainability. Country-level data and information systems played an important role in supporting the policy analysis and stakeholder mapping processes. Respondents relied on government institutions to access policy documents needed for policy analysis and, in Rwanda, used the membership of inter-sectoral working groups as a starting point for developing stakeholder lists.

*“The focal institution is the National Development Planning Commission and they put everybody awake. If we needed specific units, they helped us in getting them. We used the government machinery into this, and we were part of it. We channelled all our requests through the Planning Commission, they sent letters on our behalf for engagements and other things and that facilitated it. For the policy documents, they also helped us to get them because they themselves keep quite a lot of policy documents. They have regular meetings with the stakeholders and ministries and we used that as a springboard to get documents, the documents were not a challenge.”* (KII, academic, Ghana).

### Involvement of local experts

Country-based experts were crucial for navigating governance, accessing stakeholders, and retrieving documents. Through their professional networks, country-based experts supported access to stakeholders for interviews and meetings, as well as to relevant data and other information required for different project activities. Their familiarity with the political and governance contexts of participating countries also enabled them to navigate local systems more effectively.

*“The thing that worked well was the engagement of high-level personnel within the government. I think that worked well because the invitation to the meetings for these guys was channelled somehow through a local partner’s office in Malawi. And I think the local partner’s office team already have convening power within the country. So, I think also with the support of the local partner, I think that worked really well.”* (KII, non-government project team member, Malawi).

### Multi-stakeholder forums and intersectoral structures

Existing government working groups and dialogue platforms provided entry points for engagement, facilitating coordination across sectors. Embedding FS-TIP within these structures increased acceptability and strengthened the potential for sustained uptake.

### Government support and institutional ownership

Where government institutions actively facilitated data access and stakeholder mobilization, implementation was smoother. These findings highlight the importance of ownership by in-country institutions, reinforcing that local governance engagement is a cornerstone for successful multi sectoral initiatives.

### Participant recommendations for possible future replication of the FS-TIP approach

#### Sensitization and more early-phase networking interactions

Strong commitment from senior leadership was considered essential at the early stages of a process such as FS-TIP. Participants emphasized the importance of either increasing knowledge and awareness of the process or ensuring sufficient engagement to deepen understanding. Such efforts were viewed as critical for securing buy-in from decision-makers, which could have strengthened commitment and positively influenced implementation.

*“So, for us step one was to engage at that level, gain their buy-in, and sort of secure commitment to support the FS-TIP process but that didn't happen for a variety of reasons. It's almost like we didn't have the time because we wanted to have this analysis, these outputs ready before the summit that was part of the timing and the plan. So, by the time we were able to launch, we just had to start the work. So, I think it would have been much easier if we had had that explicit commitment from the top level to unlock data, especially from the government.”* (KII, project management office member).

Participants valued the inclusion of experts from their own countries in the implementation process. One country manager emphasized the importance of engaging experts who are physically based and residing within the country, as this could strengthen networking opportunities and local connections. Another recommendation was the establishment of a local “think tank” institution to continue supporting food systems work after completion of the FS-TIP process. Experts also underscored the need for African countries to strengthen the capacity of statistical agencies and other institutions responsible for data collection and management.

*“I thought we could also depend on administrative data, so that we don't depend heavily on surveys, because surveys are so costly, and you cannot do them every now and then. But if we have reliable routine data on almost everything we can glean, and pick out what we need. And I think that will be very useful.”* (KII, government official and project team member, Ghana).

## Discussion

This study sought to evaluate the FS-TIP initiative by examining the extent of multi-sectoral stakeholder engagement in FS-TIP activities, assessing perceived relevance and preparedness for adoption, identifying factors that facilitated or constrained implementation, and summarizing participants’ recommendations for potential future replication of the initiative. Interpreted through the Consolidated Framework for Implementation Research (CFIR) and the Intersectoral Governance Framework, the findings provide insight into how integrated food systems policy processes function as both implementation and governance interventions in low- and middle-income country contexts.

From a CFIR perspective, the perceived relevance and adaptability of the FS-TIP initiative emerged as key implementation determinants. The study participants considered the initiative relevant because its structure and mechanism enabled them to understand the key food system parameters in their countries and identify missing food system indicators that can guide future research and policy development. Participants who worked on or were involved in the process found it effective which highlights the relative success of FS-TIP as a science policy interface [22]. Familiarity with the “food system approach” central to the FS-TIP process, made the process more adaptable by the target countries, an indication that in-country dialogues are pivotal for the uptake of such a process [16, 22].

While stakeholders expressed a strong willingness to adopt the outputs of the FS-TIP process, this willingness does not automatically translate into actual adoption or institutionalization of the approach within national policy frameworks. Within CFIR, willingness reflects individual-level perceptions of relevance, acceptability, and openness to change, rather than concrete evidence of adoption. The finding highlights an important intermediate stage in the policy adoption pathway, one that reflects awareness and positive attitudes toward FS-TIP but still requires enabling structures, political commitment, and resource allocation for full implementation.

This distinction between willingness and adoption underscores the implementation gap that often exists in multisectoral policy processes, particularly in low- and middle-income countries where competing priorities, limited institutional capacity, and fragmented governance mechanisms constrain the translation of innovative approaches into practice [14, 16]. Therefore, these findings contribute to the literature by identifying willingness as a necessary but insufficient condition for food systems transformation. They point to the need for institutional anchoring, sustained capacity building, and iterative engagement to move from intent to action. Embedding FS-TIP outputs into existing national planning cycles and budgetary frameworks, for example, would be key to converting expressed willingness into tangible policy change.

The Intersectoral Governance Framework provided a useful lens for interpreting patterns of stakeholder engagement observed in the FS-TIP process. The participation of government officials in the planning and execution of FS-TIP was important for supporting continued uptake and implementation beyond the project period, while also improving access to government-held data and policy documents [7]. Despite this, their engagement remained limited, with most government representatives primarily validating outputs and clarifying selected issues. This limited participation was partly attributed to low response rates among government officials in some countries, insufficient sensitization at the start of the process, and disruptions to in-person engagement caused by the COVID-19 pandemic. Greater involvement and feedback from these stakeholders could have generated valuable insights into how the process might be refined to better respond to country-specific contexts. Similar challenges have previously been identified as barriers to the effective functioning of multi-stakeholder platforms [7, 20, 22]. In addition, sectors such as the media, grassroots organizations, and the public had limited representation in the FS-TIP process, potentially resulting in missed perspectives on food system functioning and the implementation process overall. This aligns with studies highlighting the importance of inclusive governance structures in achieving transformative food system outcomes [15].

From an intersectoral governance perspective, the FS-TIP process cannot be seen to have been fully co-created with the participating governments and embedded within existing government initiatives which is key for the success of such an initiative [16, 22]. These challenges highlight the critical need for early and ongoing engagement to foster buy-in and co-ownership, aligning with global food systems governance literature [12, 25].

Barriers to the process included inadequate centralized databases, limited availability of indicator data, and insufficient disaggregated information to support the identification of vulnerable and marginalized populations and the monitoring of food system changes in the participating countries. Challenges in accessing policy documents in some settings further highlighted the need for improved data and records management systems, alongside greater institutional preparedness. Enhancing these systems could promote easier access to and use of available data by food system stakeholders while fostering knowledge integration across the food system [4, 27]. The fast-paced implementation of the FS-TIP process limited the amount of time available for stakeholder engagement across the three countries. While the process increased awareness and visibility of the “food system” concept in these settings, continuous sensitization on the value of this approach, particularly among government stakeholders, was necessary from conceptualization and throughout implementation. This is important because food systems science often involves competing interests, tensions, and complex trade-offs, making transparent and credible information-sharing mechanisms essential to ensure that the perspectives and concerns of all actors are adequately represented and that potential trade-offs and co-benefits are clearly communicated [22, 25]. Improved understanding of food systems can also support the design of strategies that are integrated into existing sectoral plans and monitoring frameworks [4] thereby strengthening the multi-sectoral planning and implementation needed for food systems transformation.The stakeholder mapping exercise facilitated the identification of key actors within the food system, an important step in informing policy development. Application of the inter-sectoral governance framework (McQueen 2012) helped assess the participation of different sectors in the FS-TIP process; however, this assessment was constrained by the limited involvement of certain sectors. As a result, the framework was adapted to better suit the context of the evaluation. The framework therefore had to be adopted for this purpose. Governance frameworks suggest that multi-level, adaptive, and participatory approaches are essential to foster alignment between sectors and to navigate trade-offs inherent in food systems transformation (McQueen 2012). Future FS-TIP-like processes should incorporate mechanisms for ongoing feedback, think-tanks, or advisory bodies to ensure sustainability beyond the initial project phase [12, 16].

Participants emphasized that a multi-sectoral and multi-stakeholder initiative such as FS-TIP, particularly in settings where it is unfamiliar, requires adequate engagement and sensitization from the outset to build stakeholder momentum, secure buy-in, and encourage prioritization at high-level decision-making forums [4]. Such early investment in engagement could strengthen government commitment, foster goodwill, and support the development of sustainable structures for continued implementation. These recommendations align with both CFIR and intersectoral governance theory, which emphasize readiness, institutional capacity, and sustained coordination as prerequisites for successful scaling. Future implementation of a similar process would benefit from allocating more time for broader stakeholder engagement and alignment with government priorities to strengthen ownership and long-term sustainability. Establishing a think-tank or technical support mechanism during and after implementation could also help sustain momentum and support countries in advancing this work beyond the UN Food Systems Summit, particularly in contexts where the food systems approach is relatively new. Additionally, incorporating a capacity-strengthening component for government officials, especially in data interpretation and utilization, would be valuable given the data-driven nature of FS-TIP. This could enhance the selection of relevant food system indicators, improve the availability of data for these indicators, and strengthen the use of such data to monitor changes within food systems.

The evaluation of the FS-TIP process faced several challenges, partly due to the rapid pace at which the initiative was implemented. Delays in obtaining ethics approvals and research permits across the target countries slowed the research component of the project. In addition, some experts and project personnel had been engaged on short-term consultancy contracts that had expired before the evaluation took place, making it difficult to locate and involve them because of competing commitments. However, assistance from the project management office facilitated engagement with some of these individuals. Time zone differences also posed challenges in reaching experts who had participated in the FS-TIP process.

Although the research team had access to meeting minutes and summaries from stakeholder engagement activities, the information had not been systematically documented, limiting the ability to draw comprehensive conclusions about stakeholder interactions. Data collection was conducted virtually through online surveys and interviews. While survey responses were obtained, participation rates were low, limiting the breadth of the available data.

The data we have is therefore not generalizable. These data were not considered definitive or representative; instead, they served to highlight indicative patterns that either aligned with or contrasted against the qualitative insights and the desk review findings. Phone interviews would have potentially addressed this challenge as most people tend to shy away from online surveys due to lack of interest, email-checking habits and survey structure [20].

The study reflects early implementation experiences and perceived readiness to adopt FS-TIP outputs, rather than actual long-term adoption or institutionalization of the approach. Despite these limitations, triangulation of qualitative, quantitative, and desk review data allowed for indicative insights into stakeholder engagement, feasibility, and governance considerations.

## Conclusion

The FS-TIP initiative provided a structured process for assessing food system challenges in Ghana, Malawi and Rwanda and was perceived by participating stakeholders as relevant, acceptable and adoptable. The initiative enabled stakeholders to identify key gaps in food system indicators and fostered greater understanding of multi-sectoral coordination needs. Stakeholder engagement, particularly through in-country dialogues, was critical for promoting perceived usefulness and potential adoption of the FS-TIP approach.

However, willingness to adopt FS-TIP outputs does not yet equate to formal adoption or institutionalization within national policy frameworks. Limitations in stakeholder involvement, particularly among government officials and underrepresented sectors, as well as constraints in data access and the rapid pace of implementation, highlight the need for sustained engagement, capacity strengthening, and integration into existing governance structures.

Future FS-TIP–like initiatives would benefit from early and comprehensive sensitization of stakeholders, longer engagement timelines, mechanisms for ongoing feedback, and strengthened capacity for data interpretation and use. Embedding such initiatives within government-led structures and planning cycles is likely to enhance ownership, sustainability, and the potential impact on food systems transformation.

Overall, while FS-TIP demonstrates potential as a science–policy interface to guide food system policy and planning, further work is required to translate stakeholder willingness into tangible, institutionalized policy change.

## Supplementary Information

Supplementary Material 1. Supplementary Table 1. Guiding principles for the FS-TIP process. Supplementary table 2: The participation of various stakeholders from each country in the FS-TIP process. Supplementary Table 3: Level of involvement of stakeholders and sectors. Supplementary figure 1: Participant flow chart for the quantitative and qualitative study.

## Data Availability

Data described in the manuscript, code book, and analytic code will be made available upon request pending application and approval in line with the African Population and Health Research Center (APHRC) data sharing policy. Requests can be made through APHRCs [microdata portal](https:/microdataportal.aphrc.org/index.php/catalog?_gl = 1*vxsnnp*_ga*NDc2NDgzNzkxLjE2OTI3MDAxNTY.*_ga_RVZB1C7SY0*MTcxNjU0ODkxOC4xMi4xLjE3MTY1NDg5MzAuMC4wLjA.) or through this email [datarequests@aphrc.org](mailto:datarequests@aphrc.org).

## Abbreviations

CSOs: Civil Society Organizations
FS: Food systems
FS-TIP: Food systems transformative integrated policy
IDP: Integrated development program
KII: Key informant interviews
NGOs: Non-governmental organizations
PPP: Public private partnerships

## References

[1] African Union (2014, June 26–27). Malabo Declaration on Accelerated Agricultural Growth and Transformation for Shared Prosperity and Improved Livelihoods (Assembly/AU/Decl.1(XXIII)). African Union. http://archives.au.int/handle/123456789/5527

[2] Fanzo J, Haddad L, Schneider KR, et al. Viewpoint: Rigorous monitoring is necessary to guide food system transformation in the countdown to the 2030 global goals. Food Policy. 2021;104:102163.

[3] African Union Commission (2018). Second Biannual Report of the African Union Commision on the Implementation of the Malabo Declaration on Accelerated Agricultural Growth and Transformation for shared Prosperity and Improved Livelihoods African Union.

[4] Afshin A, et al. Health effects of dietary risks in 195 countries. The Lancet. 2019;393(10184):1958–72. 10.1016/S0140-6736(19)30041-8 PMC6899507 30954305

[5] Dusingizimana, Petronille; Kazungu, Jules; Lalui, Armin; Milani, Peiman; Munanura, James; Nsabimana, Aimable; Sindi, Julius Kirimi; Spielman, David J.; and Umugwaneza, Maryse. 2022. Rwanda’s food systems transformation: A diagnostic of the public policy landscape shaping the transformation process. Rwanda SSP Working Paper 4. Washington, DC: International Food Policy Research Institute (IFPRI). 10.2499/p15738coll2.135925

[6] FAO, AUC, ECA and WFP. 2023. Africa – Regional Overview of Food Security and Nutrition 2023: Statistics and trends. Accra, FAO. 10.4060/cc8743en

[7] FAO, IFAD, UNICEF, WFP, WHO. The state of food security and nutrition in the world 2021.Transforming food systems for food security, improved nutrition and affordable healthy diets for all. Rome: FAO; 2021.

[8] Faysse N. Troubles on the way: An analysis of the challenges faced by multi-stakeholder platforms. Ecol Soc 2006;11(2), 34. https://www.ecologyandsociety.org/vol11/iss2/art34

[9] Food and Agriculture Organization of the United Nations (FAO), International Fund for Agricultural Development, United Nations Children’s Fund, World Food Programme, World Health Organization. The state of food security and nutrition in the world 2022: repurposing food and agricultural policies to make healthy diets more affordable (SOFI 2022). FAO; 2022. 10.4060/cc0639en.

[10] FS-TIP 2021. A Comprehensive Food Systems Diagnostic Approach to Inform Policymaking Toward Sustainable Healthy Diets for All. https://web-assets.bcg.com/aa/cc/82176069473fafcfa63b2ca25bf7/food-system-transformative-integrated-policy-for-sub-saharan-africa.pdf

[11] High Level Panel of Experts on Food Security and Nutrition (HLPE). (2017). Nutrition and food systems: A report by the High Level Panel of Experts on Food Security and Nutrition of the Committee on World Food Security, Rome 2017 (Report No. 12). Food and Agriculture Organization of the United Nations. https://www.fao.org/3/a-i7846e.pdf

[12] High Level Panel of Experts on Food Security and Nutrition (HLPE). (2020). Food security and nutrition: Building a global narrative towards 2030 (Report No. 17). Food and Agriculture Organization of the United Nations. https://www.fao.org/3/ca9736en/

[13] IFAD. (2022). Towards food systems transformation: Five paradigm shifts for healthy, inclusive and sustainable food systems. International Fund for Agricultural Development. https://www.ifad.org/en/web/knowledge/publication/asset/42638226 10.1007/s12571-021-01221-4 PMC8517317 34667484

[14] International Food Policy Research Institute. 2021 Global food policy report: transforming food systems after COVID-19. Washington, DC: International Food Policy Research Institute; 2021. 10.2499/9780896293991.

[15] Juma PA, Mapa-tassou C, Mohamed SF, et al. Multi-sectoral action in non-communicable disease prevention policy development in five African countries. BMC Public Health. 2018;18(Suppl 1):953. 10.1186/s12889-018-5826-6. PMC6117629 30168391

[16] Kennedy E, Webb P, Block S, Griffin T, Mozaffarian D, Kyte R. Transforming food systems: the missing pieces needed to make them work. Curr Dev Nutr. 2020;5(1):177. 10.1093/cdn/nzaa177. PMC7810509 33501403

[17] Leeuwis C, Boogaard B, Atta-Krah K. Governance and food system transformations. Agric Hum Values. 2021;38:1–18. 10.1007/s10460-021-10211-0. PMC8211938 34178184

[18] Matchaya G, Nhlengethwa S, Chilonda P. Agricultural sector performance in Malawi. Reg Sect Econ Stud. 2014;14(2):141–56.

[19] Morgan R, Sonnino R, Jablonski B. Multi-stakeholder governance for sustainable food systems. Glob Food Sec. 2021;28:100485.

[20] Popkin BM. Nutrition transition and the global obesity epidemic. Nutr Rev. 2017;75(Suppl 1):3–13.

[21] Saleh A, Bista K. Examining factors impacting online survey response rates in educational research: perceptions of graduate students. J MultiDiscipl Eval. 2017;13(29):63–74.

[22] Sartas M, et al. Factors influencing participation dynamics in research for development interventions with multi-stakeholder platforms: a metric approach to studying stakeholder participation. PLoS ONE. 2019;14(11):e0223044–e0223044. 10.1371/journal.pone.0223044 PMC6855456 31725717

[23] Singh BK, Arnold T, Biermayr-Jenzano P, et al. Enhancing science–policy interfaces for food systems transformation. Nat Food. 2021;2:838–42. 10.1038/s43016-021-00406-6. 37117505

[24] SOFI. State of food security and nutrition in the world. FAO; 2025.

[25] Turnhout E, et al. Do we need a new science-policy interface for food systems? Science. 2021;373(6559):1093–5. 10.1126/science.abj5263 34516868

[26] Water for Sustainable Food and Agriculture, Food and Agriculture Organization of the United Nations, Rome, 2017

[27] Wilkinson M, Dumontier M, Aalbersberg I, et al. The FAIR guiding principles for scientific data management and stewardship. Sci Data. 2016;3:160018. 10.1038/sdata.2016.18. PMC4792175 26978244

[28] World Bank national accounts data, and OECD National Accounts data files 2018. https://data.worldbank.org/indicator/NV.AGR.TOTL.ZS. Accessed 29 Mar 2022.

