# Supplementary material for: Enablers and barriers to the implementation of the Food System Transformative Integrated Policy (FS-TIP) in Ghana, Malawi and Rwanda
Source: BMC Public Health. 2026 Aug 1;26:3328. doi: 10.1186/s12889-026-28169-4 (PMC13753151; doi:10.1186/s12889-026-28169-4)
Supplement: Supplementary file 1 [file 12889_2026_28169_MOESM1_ESM.docx]

**Food System Transformative Integrated Policy (FS-TIP) Questionnaire for Stakeholders Engaged in the Process**

**Food System Transformative Integrated Policy (FS-TIP): Establishing the Policy Foundation and Supporting Food System Country Dialogues and Analytics.**

**Insert consent**

1. **Basic information**
   1.1 **Country:** 1. Malawi 2.Rwanda 3. Ghana
   1.2 **Gender:** 1. Male 2. Female
   1.3 **Department:**
   1.4 **Position (optional):**
   1.5 **Number of years in service: 1. 0-4 years 2. 4-8 years 3. 8-12 years 4. >12 years**
   1.6 **Sector:**	1. Health 2. Agriculture 3. Trade 4. Academia 5. Transport 6. Finance
   1.7 **Type of Organization:** 1. Government 2. Non-governmental organization 3. Private company
2. **FS-TIP Approach**

The Food System Transformative Integrated Policy, FS-TIP is a long-term endeavor aiming to contribute to food system transformation, achievement of the Sustainable Development Goals and sustainable healthy diets for all, primarily through policy. The process involves;

(i) identification of key indicators required to track food system progress;
(ii) (ii) a diagnostic and landscape analysis and mapping, which aims to identify food system policy challenges and key stakeholders in the food systems;
(iii) (iii) the development of a tool kit that can be used to implement the process in other countries.
      2.1 To what extent were you engaged in any of the steps of the FS-Tip process?

- Identification of indicators in the food system
- Policy mapping
- Stakeholder mapping
- Review of the toolkit
  2.2 What are your general thoughts about the intentions of the FS-TIP process?

(enter text)

2.3 Do you Strongly agree (1), Agree (2), Neither agree nor disagree – i.e Undecided (3), Disagree (4), or Strongly disagree (5) that the FS-TIP approach:

| **The FS-TIP Approach** | **Strongly Agree** | **Agree** | **Undecided** | **Disagree** | **Strongly disagree** |
| --- | --- | --- | --- | --- | --- |
| CODES | **5** | **4** | **3** | **2** | **1** |
| 1. Aligns with country needs |  |  |  |  |  |
| 2. Engaged relevant stakeholders from different sectors |  |  |  |  |  |
| 3. Defined food system priority areas for my country |  |  |  |  |  |
| 4. Is relevant for my country |  |  |  |  |  |
| 5. Is helpful/useful for my country |  |  |  |  |  |
| 6. Is easy to implement |  |  |  |  |  |

The FS-TIP process was guided by five principles listed in the table below. Kindly give your opinion on the process as per your participation in the process.:

| **The FS-TIP diagnostic process was guided by the following principles** | **Strongly Agree** | **Agree** | **Undecided** | **Disagree** | **Strongly disagree** |
| --- | --- | --- | --- | --- | --- |
| CODES | **5** | **4** | **3** | **2** | **1** |
| 1. **Designed with the policy-maker in mind**: presenting an interface that is concise, compelling and intuitive |  |  |  |  |  |
| 2. **Outcome oriented:** linking indicators that reflect the outcome of food systems transformation to the drivers that policy-makers can influence to realize the transformation |  |  |  |  |  |
| 3. **Anchored in existing structures:** building on existing resources and structures with strong buy-ins, such as Comprehensive Africa Agriculture Development Program (CAADP)* biennial review report and adding new elements only where required |  |  |  |  |  |
| 4. **Aligned to existing food systems frameworks:** connecting to UN FSS Action Tracks^$^ for its outcome orientation and covering all components of the food system |  |  |  |  |  |
| 5. **Tailored to Africa and country-context:** adapting indicators to the countries' context, leveraging local data sources and reflecting local ambitions (developing together where non-existent) |  |  |  |  |  |

***** **Comprehensive Africa Agriculture Development Program CAADP-**is a framework that has inspired and energised African agricultural research institutions, farmers’ associations, African governments and the private sector who believe that agriculture has a pivotal role in development. It aims to boost investment to stimulate growth in the agricultural sector. This means bringing together the public and private sectors and civil society – at the continental, regional and national levels – to increase investment, improve coordination, share knowledge, successes and failures, encourage one another and to promote joint and separate efforts.

**^$^UN FSS Action Tracks:** offer stakeholders from a wide range of backgrounds a space to share and learn, with a view to fostering new actions and partnerships and amplifying existing initiatives. Each Action Track is designed to address possible trade-offs with other tracks, and to identify solutions that can deliver wide-reaching benefits. They include: (i) Ensure access to safefoods for all; (ii) shift to sustainable consumption patterns; (iii) boost nature positive production; (iv) advance equitable livelihood; (v) Build resilience to vulnerabilities, shocks and stress

3. **What else could have been done to make the process more:**
   3.1 User friendly
   3.2 Outcome oriented
   3.3 Anchored in existing structures
   3.4 Aligned to existing food systems frameworks
   3.5 Tailored to thecountry context and Africa
4. **Landscape and diagnostic analysis, stakeholder and policy mapping**

The FS-TIP approach followed several process including identification of key indicators required to track food system progress, a stakeholder mapping exersice which aimed to identify key stakeholders required for a functional food system and a policy mapping exercise which aimed to identify policies required for proper functioning of a food system. How satisfied are you with the key steps of the FS-TIP Approach?

| **Key Steps FS-TIP Approach** | **Very satisfied** | **Satisfied** | **Neutral** | **Disatisfied** | **Very Disatisfied** |
| --- | --- | --- | --- | --- | --- |
| **CODES** | **5** | **4** | **3** | **2** | **1** |
| 1. Identification of key indicators required to track food system progress |  |  |  |  |  |
| 2. Stakeholder mapping |  |  |  |  |  |
| 3. Policy mapping |  |  |  |  |  |

4.1 What is your opinion, for each of the key processes
- What worked well and why?
  - Identification of key indicators for the food system
  - Stakeholder mapping
  - Policy mapping
- What did not work well and why?
  - Identification of key indicators for the food system Stakeholder mapping
  - Policy mapping
- What can be done to improve the process for scale up of?
  - Identification of key indicators for the food system Stakeholder mapping
  - Policy mapping

5. To what extent were outputs (Identification of key indicators for the food system, stakeholder and policy mapping):
   5.1 Relevant to your country context
       5.1.1 Identification of key indicators for the food system
       5.1.2 Stakeholder mapping
       5.1.3 Policy mapping
   5.2 Useful in understanding the current food system challenges
       5.2.1 Identification of key indicators for the food system
       5.2.2 Stakeholder mapping
       5.2.3 Policy mapping
   5.3 Representative of the actual situation on the ground
       5.3.1 Identification of key indicators for the food system
       5.3.2 Stakeholder mapping
       5.3.3 Policy mapping
   5.4 Are you willing to adopt? If yes, why, if no, why not?
       5.4.1 Identification of key indicators for the food system
       5.4.2 Stakeholder mapping
       5.4.3 Policy mapping
   5.5 Are you ready to adopt? If yes, why, if no, why not?
       5.5.1 Identification of key indicators for the food system
       5.5.2 Stakeholder mapping
       5.5.3 Policy mapping
6. **The Toolkit**

The final output of the FS-TIP process was a food systems analysis tool kit which aims to guide all those interested in food systems to conduct a systematic, thorough and comprehensive diagnosis and landscaping analysis of the food system of a country by using a set of tools. It offers step-by-step processes as well as practical tools to help users conduct a food systems analysis. It enables users to build a foundation of facts using a human-centered design approach. The results from the diagnostic and landscaping analysis can then form the basis for selecting priority challenges to address, setting ambitious targets and developing and implementing effective policies and programs that leverage synergies and account for trade-offs.

How satisfied are you with:

| **Key Steps FS-TIP Approach** | **Very satisfied** | **Satisfied** | **Neutral** | **Disatisfied** | **Very Disatisfied** |
| --- | --- | --- | --- | --- | --- |
| **CODES** | **5** | **4** | **3** | **2** | **1** |
| 1. Tools provided for the Identification of key indicators for the food system |  |  |  |  |  |
| 2. Policy landscaping tool |  |  |  |  |  |
| 3. Stakeholder mapping tool |  |  |  |  |  |

6.1 To what extent is the toolkit?
    6.1.1 Relevant to your country
    6.1.2 Sufficiently inclusive of:

Policy makers and technical experts within government willing to champion or promote food system transformation

Development partners, academics and experts who want to support stakeholders to better understand the current status of their food system and how to transform it

6.1.3 Acceptable to your country processes
6.1.4 Adoptable in your country
6.2 What can be done to make the tool kit more
    6.2.1 Relevant in your counry context
    6.2.2 Inclusive better participation in your country
    6.2.3 Acceptable within your country context and processes
    6.2.4 Adoptable in your country processes and frameworks.

**Qualitative questionnaire**

**FOOD SYSTEM TRANSFORMATIVE INTEGRATED POLICY: Establishing the Policy Foundation and Supporting Food System Country Dialogues and Analytics**

**Process Stages Qualitative tool**

**1.** **Process: High level rapid assessment – to interview those conducting it for each country**

Ask a question to the desk reviewers, and others people engaged, more specific questions on their engagements.

·	What has worked well in the rapid assessment?

·	What was challenging during this process? Why?

·	How could have been done differently for this kind of high level rapid assessment?

**2.** **Process stage: landscape analysis for each country – to interview those overseeing and conducting the country landscape analysis.**

·	What was the landscape analysis experience like? Why do you say so?

·	What worked well during this task?

·	What was challenging during this task? Why do you say so?

·	What could have been done differently?

·	What facilitated your ability to map out food system players in your respective country?

·	What facilitated your ability to identify FS related disagreements in your respective country.

**3.** **Deep-dive assessments on country priority areas: Interview some of those who participated in the assessment and those who coordinated this exercise**

**Participants & coordinators**

·	How was the process of generating challenges and opportunities in the food system in your country carried out?

·	What worked well in this process? What else facilitated the success of this process?

·	What did not work well? Why do you say so?

·	What challenges did you experience in this process?

·	What would you recommend be done differently in a similar process?

**Co-ordinators**

·	Which of your expectations for this process was not met?

**4.** **Country dialogues towards sharing outputs at UN-FS: Interview Country point people and those coordinating the dialogues**

·	What was your experience with the in-county dialogues?

·	How were the dialogues carried out? How many meetings were they?

·	What worked well through this dialogues?

·	What did not work well during this dialogues? Why do you say so?

·	What challenges did you experience?

·	What would you recommend be done differently when scaling this process to other countries?

·	How useful were the country dialogues and process in helping country actors assimilate these outputs (country level dialogues) into their agricultural plans and other country ministries and organs?

·	How did you find the process, what worked well, what could have been done differently?

**5.** **Transitioning beyond the FS-TIP pilot**

**Country Coordinators**

·	In your view, what has worked well to transition the in-country teams to further this work?

·	What gaps need to be filled in order to keep momentum and intended outcomes?

·	What remains a challenge at this point?

·	What could have been done differently to ensure a better transition for the country teams?

**In-country coordinators**

·	How the entire engagement from the start up until this point helped your country team to keep momentum and engagements around the food sytem?

·	What continues to facilitate this momentum and engagement?

·	What has been a challenge for you?

·	What would you recommend be done differently in other countries?

·	What other stakeholders should have been considered for engagement through this process?

**6.** **PMO Coordinators to assess their experience across the stages:**

·	What has been your experience in coordinating and steering and managing this collaboration? Why do you say so?

·	What has worked well?

·	What has facilitated your successes in the entire process of coordination activities across the three countries?

·	What has not worked well? Why?

·	What would you recommend continue to be done as FS-TIP is extended into other countries?

·	What would you recommend be done differently as FS-TIP be extended to other countries?
